# Mistaken identity: Reporting two cases of rare forms of extrapulmonary tuberculosis in Solomon Islands

**DOI:** 10.1016/j.ijscr.2023.109141

**Published:** 2023-12-10

**Authors:** Dylan Bush, Florence Fiuramo, Jahrad Liligeto, Lydia Ipulu, Jason Diau, Rooney Jagilly

**Affiliations:** Ministry of Health & Medical Services, Honiara, Solomon Islands

**Keywords:** Tuberculosis, Solomon Islands, Limited-resource, Pott disease, Case report

## Abstract

**Introduction and importance:**

Extrapulmonary tuberculosis (EPTB) is a relatively rare and difficult-to-diagnose manifestation of *Myobacterium tuberculosis* (TB) infection.

**Case presentation:**

This study reports the cases of a 47-year-old male and a 35-year old female with rare forms of EPTB who sought medical care in Solomon Islands. Both patients presented with nondescript symptoms and a chief complaint of pain. Initial diagnosis for the male and female patient was an abacterial colon polypoid mass and a urinary tract infection (UTI) respectively. Following unsuccessful treatment for UTI and further investigation, the surgical team diagnosed the female patient with a tuberculosis spondylitis and a bilateral psoas abscess. The male patient was subsequently diagnosed with isolated colonic tuberculosis. After starting medication, the patients were discharged and prescribed 9-month treatment regimens. During outpatient treatment both patients reported suboptimal adherence. The female patient resumed treatment and showed improvement while the male patient discontinued treatment, experienced worsening symptoms, and ultimately died.

**Clinical discussion:**

The nonspecific symptoms of extrapulmonary TB infection make it difficult to diagnose. Cases of rare forms of EPTB are particularly challenging to identify. Misdiagnosis may further increase the likelihood of mortality and morbidity in these cases. Intensive medication counseling, patient outreach, and regularly scheduled follow-up visits may reduce the incidence of poor adherence and reduce the risk of developing drug-resistant TB.

**Conclusion:**

Medical practitioners in tuberculosis-endemic countries like Solomon Islands should maintain a high clinical index of suspicion in diagnosing EPTB. Future research should investigate the prevalence of TB and EPTB in the Solomon Islands.

## Highlights

• Extrapulmonary tuberculosis (EPTB) may present with varied and nondescript symptoms.
• Bilateral psoas tuberculosis (TB) and isolated colonic TB are exceedingly rare.
• Limited access to diagnostic resources further delays EPTB diagnosis and treatment.
• Without proper treatment EPTB can cause life-altering disability and death.
• Proper medication adherence is key to effective EPTB treatment.

## 1 Introduction

Although extrapulmonary tuberculosis (EPTB) has historically been a rarer manifestation of *Myobacterium tuberculosis* (TB) infection, its relative frequency has increased in recent decades [1,2]. This condition is primarily found in children and immunocompromised individuals and is rarer in immunocompetent adults [3,4]. The threat of EPTB is particularly great in tuberculosis-endemic countries and resource-limited contexts where the high burden of disease combined with limited health infrastructure can delay detection and treatment [5,6].

Solomon Islands, a medium burden TB country, has an estimated incidence of approximately 72 cases of TB per 100,000 population [7]. Within the Pacific region, Solomon Islands is categorized as one of six countries with a high burden of TB [8]. The limited health surveillance and data collection infrastructure in Solomon Islands may contribute to underreporting of new TB cases [9].

Although tuberculous spondylitis or Pott's disease is the most common form of extrapulmonary tuberculosis, Pott's disease with bilateral psoas abscess is exceedingly rare [10,11]. Similarly, isolated colonic tuberculosis is one of the rarest manifestations of the disease [12]. To the author's knowledge these are the first reported cases of Pott's disease with bilateral psoas abscess and isolated colonic tuberculosis in Solomon Islands and more broadly in a Pacific Island country (PIC). We hope that these cases will draw attention to the risk of EPTB in TB-endemic countries like Solomon Islands and will encourage clinical consideration of EPTB in cases of chronic abscesses.

This work has been reported in line with the SCARE guidelines [13].

## 2 Case presentation

### 2.1 Case 1

A 34-year-old Melanesian female presented to the emergency department with normal vital signs, flank pain, and low-grade fever. The patient had a history of a Bartholin abscess, right ovarian cyst, and self-resolved pleural effusion within the past year but no history of TB infection. The patient was treated outpatient for a suspected UTI within the past year with a standard regimen of nitrofurantoin (100 mg 2×/day), metronidazole (400 mg 3×/day), with acetaminophen, and ibuprofen for pain management. Nevertheless, back pain persisted for 7 months. On re-admission, the patient was referred to gynecology and was again treated outpatient for a suspected UTI with trimethoprim (300 mg 1×/day) and acetaminophen for pain. When the patient returned 10 days later with persistent bilateral flank pain, her medical team adjusted her treatment regimen to amoxycillin (500 mg 3×/day), metronidazole (400 mg 3×/day), nitrofurantoin (100 mg 2×/day) and acetaminophen. Additionally, an ultrasound of the abdomen and kidneys was ordered, and the patient was referred to the surgical team.

When the patient presented to the surgical ward to review her ultrasound results, her flank pain had spread to include the upper lumbar region (L1 & L2). Ultrasound revealed complex collection (10.5 × 4.8 × 7.4 cm) inferior to the right kidney with loss of the normal proximal aspect of the right psoas muscle. A follow-up CT of the abdomen pelvis was scheduled, and the patient was placed on a regimen of cloxacillin (500 mg 4×/day), ibuprofen, and acetaminophen. The patient independently sought out *kastom* medicine, a traditional set of herbal and spiritual medical practices in Solomon Islands, before returning for her CT two months later.

**Fig. 1:**
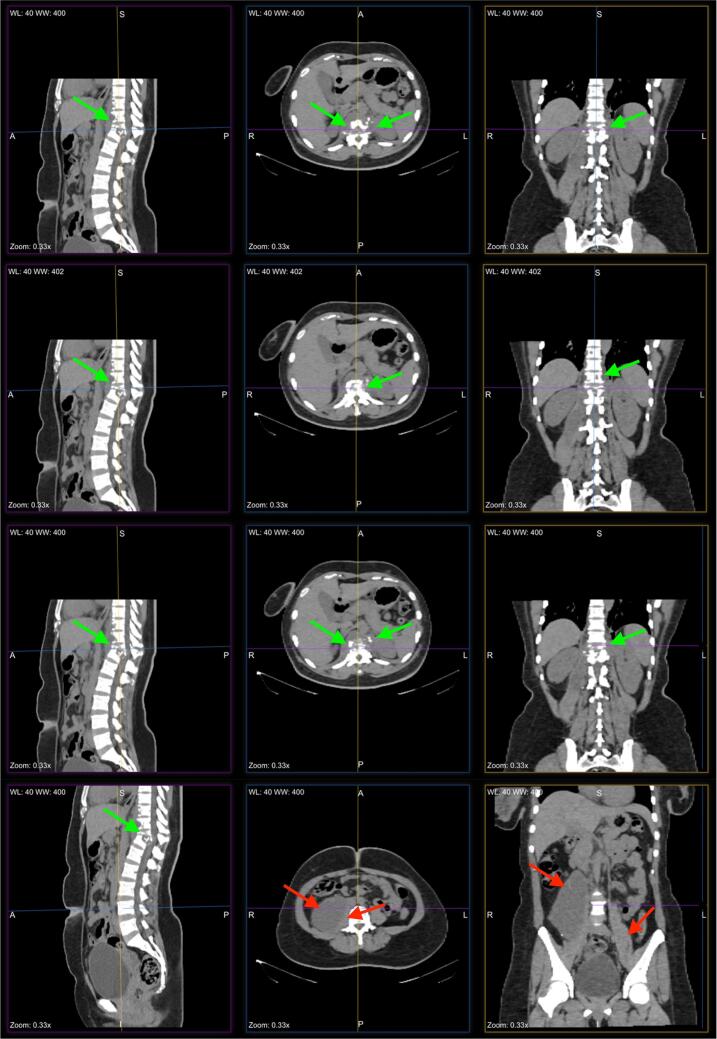
Computerized tomography scan of female patient showing bony destruction of the T8-T12 vertebrae as well as loss of height and kyphosis of T11. A paravertebral abscess can be seen extending from T8 to L1 level. The right psoas abscess is more easily appreciated on CT due to its large size (5 × 4.3 × 15.3 cm) as compared to left-side psoas abscess (1.1 × 1.3 × 3.4 cm). Key features including bilateral psoas abscesses and bony destruction of the spinal column have been identified with red and green arrows respectively. (For interpretation of the references to colour in this figure legend, the reader is referred to the web version of this article.)

Radiologic imaging (Fig. 1) revealed bony destruction of the T8-T12 vertebrae with a loss of height and kyphosis of T11. Of further concern, bone and inflammatory debris of T11 was noted to be encroaching into the spinal canal at the T11-T12 level. Lytic lesions were also visualized on the L1 and L2 vertebrae. A paravertebral abscess was seen extending from T8 to L1 and a bilateral psoas abscess was visualized. The psoas abscess was irregular in distribution, measuring 5 × 4.3 × 15.3 cm on the right side and 1.1 × 1.3 × 3.4 cm on the left. Additionally, focal omental lymphadenopathy was seen in the right lower quadrant. The limited view of the lungs (below T6) showed septal thickening and consolidation in the right middle lobe. A functional right ovarian cyst (3.3 × 2.8 cm) was also noted.

A surgical registrar performed an incision and drainage (I&D) of the psoas abscess with tubes inserted bilaterally for continuous drainage. A total of 10 mL of straw-colored fluid were drained from the right side with minimal collection (<2 mL) drained from the left abscess over two days. The surgical team collected a sample of fluid from the right psoas abscess for laboratory analysis. When this sample did not culture, the surgical team suspected TB and screened the patient for TB exposure. Further questioning revealed that the patient had been the primary caretaker for her mother during an infection of pulmonary tuberculosis four years prior. Subsequent mycobacterial testing was positive, and the patient was diagnosed with Pott's disease involving a bilateral psoas abscess. Importantly, chest x-ray was unremarkable in this case. The patient was discharged and placed on a nine-month treatment of HRZE^1^ with pyridoxine. On six-week follow-up the patient reported moderate resolution of flank pain and was observed to have permanent kyphosis. The patient acknowledged occasionally forgetting to take her medication.

### 2.2 Case 2

**Fig. 2:**
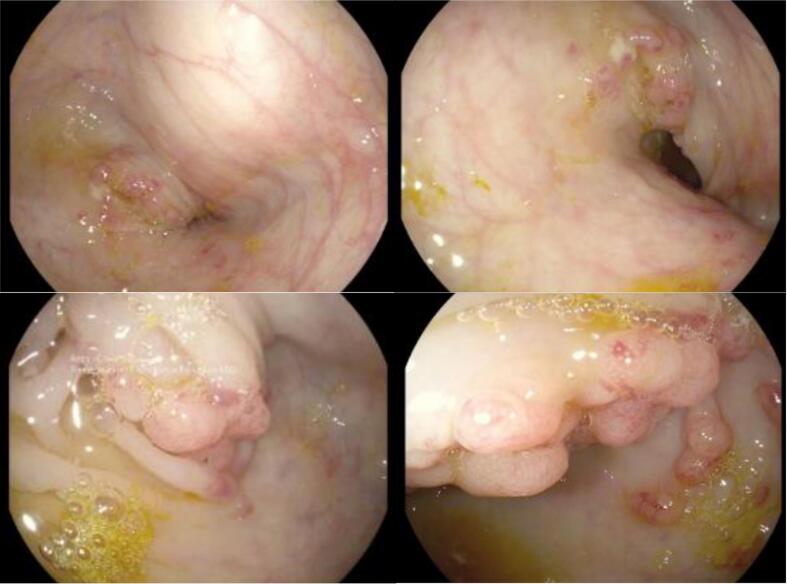
Colonoscopy of male patient showing a distal ascending colon polypoid mass extending into the lumen of the ascending colon.

A 47-year-old Melanesian male presented to the emergency department following referral from a provincial hospital for symptoms including moderate grade fever (39 °C), decreased appetite, bloody stool, severe and ascending abdominal pain, and generalized weakness over the previous week. The patient reported inability to pass stool over the previous two days. Two months prior, a colonoscopy had revealed a right ascending colon mass that extended into the intestinal lumen (Fig. 2). Biopsies were taken at that time, but the samples were lost in transit to the Australia-based laboratory.

**Fig. 3:**
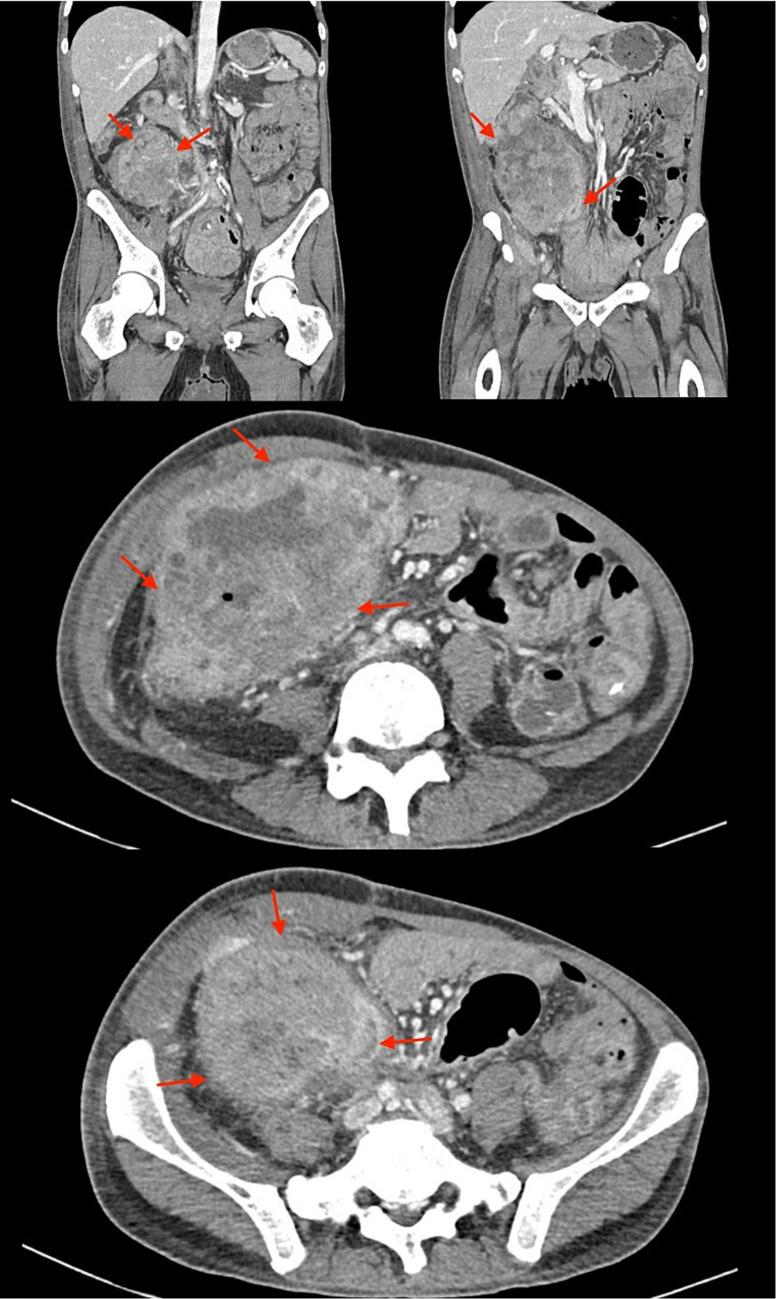
Computerized tomography scan of male patient showing a large complex mesenteric mass of the ascending colon that obliterates the appendix. Bowel wall thickening in the lower abdominal quadrants can also be appreciated. The mass has been identified with red arrows. (For interpretation of the references to colour in this figure legend, the reader is referred to the web version of this article.)

On readmission, a computerized tomography scan was performed and showed a complex mesenteric mass of the ascending colon without abdominal lymphadenopathy or metastasis (Fig. 3). The surgical team was consulted and suspected that the mass was the result of colon cancer. Biopsies were collected and sent to Australia for histopathological analysis. The patient was instructed to return in 4 weeks to the medical ward to review results. Histopathological results indicated tissue changes consistent with an inflammatory polyp including mild crypt irregularity, an increase in mixed inflammatory cells in the lamina propria, and some focal surface erosion. Notably, there was no evidence of dysplasia or malignancy. At follow-up, the patient reported worsening pain and was readmitted. Vitals were normal apart from a blood pressure of 90/50 mmHg and a respiratory rate of 22 breaths per minute. On palpation of the right iliac fossa, the patient exhibited abdominal distension, a palpable mass, tenderness, and guarding. Bloodwork on admission is listed in Table 1. The patient was admitted to the medical ward and an intravenous (IV) saline drip was initiated along with cloxacillin (1 g 4×/day), metronidazole (500 mg 3×/day), with oral acetaminophen (1 g 4×/day) and morphine (3 mg pro re nata) for pain.

**Table 1:** Bloodwork on admission for male patient.

Test type	Result
Hemoglobin (g/L)	109 (110–180)
Erythrocyte (x 10^12^/L)	4.36 (3.9–5.1)
MCV (fL)	71.8 (84–94)
MCH (pg)	25.0 (27–34)
MCHC (g/L)	348 (300–350)
White Blood Cell Count (x 10^9^/L)	28.49 (4.0–11.0)
Neutrophil (%)	90.9 (40–75)
Lymphocyte (%)	3.3 (20–45)
Monocyte (%)	5.5 (2−10)
Platelets (x 10^9^/L)	581 (150–400)
Sodium (mmol/L)	124 (135–146)
Potassium (mmol/L)	4.6 (3.5–4.6)

Given the patient's worsening symptoms, he was again referred to the surgical ward for inpatient management and further investigation. Considering the histopathological results and the patients' symptoms, the surgical team suspected a colonic abscess of bacterial etiology and thus added IV ceftriaxone (1 g 2×/day), oral Fefol^2^ (150.5 mg 1×/day), and switched the patient to IV morphine (3 mg 4×/day). Further bloodwork, ultrasound, I&D, and a repeat colonoscopy was ordered. Pus collected during I&D cultured both gram positive cocci and gram-negative bacilli. The laboratory identified clindamycin and erythromycin-resistant *staphylococcus aureus* within the culture. Ultrasound confirmed the presence of a heterogenous right ilia fossa mass (10.6 × 8.3x10cm). Repeat colonoscopy revealed a polypoid mass in the ascending colon that obstructed the cecum (Table 1). The gastroscopy team again sent samples for histology. A subsequent exploratory laparotomy allowed the surgical team to palpate a firm intra-abdominal mass with attachment to the small bowel and greater omentum, making surgical removal difficult. A surgical registrar placed a drain in situ to remove fluid from the abscess.

When histology results from the colonoscopy showed no evidence of granulomatous inflammation, dysplasia, or malignancy and instead were consistent with an inflammatory polyp, pus was collected for culturing and nucleic acid amplification TB testing. Microbiology results cultured mixed enteric flora, *Escherichia coli* positive for extended spectrum beta-lactamase, and klebsiella pneumonia that was resistant to ampicillin, amoxicillin, Augmentin, ceftriaxone, cotrimoxazole, and gentamicin. TB testing was positive. Following receipt of these results, the patient was diagnosed with an extrapulmonary TB infection isolated to the colon. The patient was placed on a nine-month HRZE regimen with Fefol, vitamin B_6_, and acetaminophen for pain. The patient was managed outpatient due to a limitation of bed space in the TB ward. Despite initial improvement during inpatient treatment, the patient returned within two weeks of discharge and reported defaulting on medication. The patient was re-admitted with low-grade fever, nausea, vomiting and reduced appetite. Despite attempted re-initiation of therapy, the patient decompensated and died within one month of re-admission.

## 3 Discussion

The cases presented above demonstrate the threat posed by extrapulmonary tuberculosis. Not only are the diverse presentations of EPTB difficult to diagnose, but its relative increase in frequency coupled with the increasing rate of TB infection in the Pacific region make EPTB particularly dangerous in Solomon Islands [14]. Furthermore, the increased prevalence of diabetes in the region may increase the immunosusceptibility of local populations to life-threatening infections like EPTB [15,16].

Misdiagnosis was an important factor that delayed treatment in the cases presented above. Importantly, misdiagnosis of rare forms of EPTB occurs in both developing and developed nations [[17], [18], [19]]. EPTB infection in organs with a high normal burden of bacterial flora, like the colon, can be particularly difficult to diagnose as samples may culture pathogenic bacteria that disguise the mycobacterial tuberculosis infection, delaying diagnosis. Thus, prompt and aggressive management, including early and minimally invasive abscess drainage as well as mycobacterial testing when feasible, could contribute to more rapid diagnosis in cases of EPTB.

In both cases presented here, patients had symptoms for longer than two months, with one patient suffering from severe pain for more than one year. Importantly, both sociocultural and infrastructural factors also contributed to delays in these cases. In the case of the female patient, when conventional urinary tract infection treatments failed, she became frustrated and sought out alternative, non-biomedical healing, delaying the detection and treatment of her EPTB as the infection spread dangerously close to her spinal cord.

The lack of a functional medical records system and laboratory outsourcing further delayed treatment decisions and diagnosis. Without accessible medical records, doctors were unable to comprehensively review prior medical history in these cases. In the case of the female patient, this led to repeated treatment for suspected UTI despite non-response. Furthermore, histopathology specimens from Solomon Islands must be sent to Australia for processing. Samples may be lost or damaged during transit and wait times for results often exceed three weeks. There is an urgent need to build infrastructure that enables local histopathological processing.

Both patients demonstrated suboptimal medication adherence. In the case of the male patient, therapy default resulted in the worsening of his condition and ultimately to the patient's death. The unavailability of inpatient beds in the TB ward made daily observed therapy impossible in both cases. Further research should investigate alternative community-based methods to ensure medication adherence.

In order to prevent such delays in effective anti-TB treatment in the future, it will be important to rapidly recognize EPTB not only in tertiary care centers, but also in the field. Point-of-care ultrasound has been shown to potentially offer an effective and feasible method to quickly identify EPTB in resource-limited contexts [20]. In countries with endemic TB like Solomon Islands, the implementation of community-based screening and standardized clinical questionnaires to identify TB risk may hold promise, especially in the identification of EPTB. Furthermore, these cases demonstrate that the creation of locally-tailored diagnostic guidelines for extrapulmonary masses of unknown etiology, with particular attention to prompt mycobacterial testing, may improve treatment outcomes in cases of EPTB. We hope these cases serve to alert medical practitioners and researchers to the threat of EPTB in Solomon Islands and in the Pacific region more broadly.

## 4 Conclusion

In conclusion, we presented two rare manifestations of EPTB in which the infection was initially unrecognized, and the patients were initially treated for other conditions. In both cases, a combination of surgical, radiological, and laboratory investigations eventually led to a conclusive EPTB diagnosis. A high clinical index of suspicion coupled with more detailed history-taking and point-of-care diagnostics may help to reduce morbidity and mortality in these cases.

## Consent

Written informed consent was obtained from the patients for publication of this case report and accompanying images. A copy of the written consent is available for review by the Editor-in-Chief of this journal on request.

## Provenance and peer review

Not commissioned, externally peer-reviewed.

## Ethical approval

Ethical approval was provided by the Solomon Islands Health Research Ethics Review Board (SIHRERB), Honiara, Solomon Islands (Project number HRE025/23).

## Funding

No funding received.

## Credit authorship and contribution statement

Dylan Bush – Primary author of case report *(*Conceptualization, drafting of manuscript).

Dr. Florence Fiuramo – Assisting surgeon on bilateral psoas abscess case (case note interpretation, clinical data procurement, interpretation).

Dr. Jahrad Liligeto - Lead surgeon on colonic tuberculosis case, editor (case note interpretation, imaging data procurement).

Dr. Lydia Ipulu - Assisting surgeon on colonic tuberculosis case (case note interpretation, data procurement).

Dr. Jason Diau – Lead surgeon on bilateral psoas abscess case.

Dr. Rooney Jagilly – Consultant surgeon on both cases (Case analysis, revision of prior drafts, final approval of manuscript).

## Research registration

N/A.

## Guarantor

Dylan Bush.

## Declaration of competing interest

The authors report no declaration of competing interest.

## Footnotes

1 Isoniazid (5 mg/kg), rifampicin (10 mg/kg), pyrazinamide (25 mg/kg), pyridoxine (25 mg), and ethambutol (15 mg/kg) for 2 months followed by isoniazid (5 mg/kg), rifampicin (10 mg/kg), and pyridoxine (25 mg) for 7 months.

2 Fefol is a brand name supplement that includes a mix of 150 mg ferrous sulfate and 500 μg folic acid.
